# A Rare Presentation of Advanced‐Stage Low‐Grade Serous Carcinoma in a 27‐Year‐Old Patient. Navigating a Complex Postoperative Course: Submassive Pulmonary Embolism and a Large Retroperitoneal Haematoma Following Radical Ovarian Cytoreductive Surgery

**DOI:** 10.1002/ccr3.71430

**Published:** 2025-11-06

**Authors:** Hannah Williams, Sabina Ioana Nistor, Slaveya G. Yancheva, Nicholas Symons, Hooman Soleymani majd

**Affiliations:** ^1^ University of Oxford Medical School Oxford UK; ^2^ Department of Gynaecological Oncology, Churchill Hospital Oxford University Hospitals National Health Service (NHS) Foundation Trust Oxford UK; ^3^ Department of Cellular Pathology Oxford University Hospitals Oxford UK; ^4^ Department of Colorectal Surgery Oxford University Hospitals NHS Foundation Trust Oxford UK; ^5^ Nuffield Department of Women's and Reproductive Health University of Oxford Oxford UK

**Keywords:** carcinoma, low‐grade, ovarian epithelial, pulmonary embolism

## Abstract

Ovarian Low Grade Serous Carcinoma (LGSC) is a rare gynecological cancer with limited representation in literature. Achieving R0 through maximal cytoreductive effort is indicated to provide the best outcome. Gynecological cancer surgery increases risk of pulmonary embolism—balancing this with bleeding risk complicates the post‐operative landscape.

## 1 Background

Ovarian cancer is an aggressive gynecological malignancy, with global cancer burden data from 2022 attributing it to 4% of female oncology deaths worldwide [1]. Due to the typically non‐specific presentation of bloating and abdominal discomfort it is frequently detected at advanced International Federation of Gynecology and Obstetrics (FIGO) stages. Most ovarian tumors are epithelial, with the most common ovarian epithelial tumors being serous. These are further subtyped as benign, serous borderline tumors (SBTs), low‐grade serous carcinomas (LGSCs) and high‐grade serous carcinomas (HGSCs). HGSCs account for approximately 70% of serous neoplasms [2, 3]. LGSCs are rarer, accounting for ~2% of all epithelial ovarian cancers and 4.7% of serous ovarian cancers [4]. They are characteristically insidious, arising *de novo*, from benign tumors or from SBTs [3, 5, 6]. LGSCs show distinct cytological and genetic differences to HGSCs. Like other rare epithelial ovarian cancers such as clear cell carcinoma and mucinous adenocarcinoma [7, 8], LGSCs respond little to chemotherapy [3, 9].

This case report describes a nulliparous woman in her late 20s who presented with severe abdominal pain and bloating. Cytology of ascitic fluid identified atypical cells consistent with a serous tumor without high‐grade malignant features. On imaging, large bilateral ovarian masses and evidence of omental disease were suggestive of FIGO stage IIIC, and surgery confirmed spread to the uterus, rectum, spleen, appendix, peritoneum and right hemidiaphragm. She has consented for her case to be published. This case illustrates a multidisciplinary effort to perform primary/upfront radical ovarian cytoreductive surgery in a maximal surgical effort to remove all macroscopic disease (achieve R0) in a young patient with a chemo‐resistant ovarian tumor, and to safely navigate a postoperative course complicated by both thromboembolism and bleeding.

## 2 Case Presentation

Whilst traveling in Australia, this 27‐year‐old nulliparous patient presented to local healthcare services with abdominal bloating, fatigue and early satiety. She had a medical history of post‐COVID asthma, anxiety, depression and a BMI of 32, with no significant surgical or gynecological history. Initial imaging by Australian colleagues indicated bilateral ovarian masses and subtle changes suggestive of omental metastases, but no indication of lymph node involvement. A CT chest showed no evidence of metastasis. Aspiration and histological analysis of ascitic fluid indicated a p53 wild type (WT) LGSC. As treatment in Australia would require the patient to stay for up to 1 year, she opted to receive treatment in the UK with familial support. In Oxford, large bilateral masses were palpable to the xiphisternum, and she was urgently referred for multidisciplinary team (MDT) discussion.

## 3 Investigations

At the MDT, ovarian cancer marker Ca‐125 was 24,000 ku/L, whereas other tumor markers (CEA, Ca19.9, LDH, bHCG and aFP) were all normal.

A repeat CT Chest/Abdomen/Pelvis confirmed the presence of bilateral complex cystic adnexal masses, with the right measuring 29 cm and the left 22 cm at maximum diameter. Subtle peritoneal nodularity was noted alongside omental stranding and nodularity, suggestive of malignant change. Linear calcification on the anterior aspect of the uterus indicated involvement. No lung nodules, pleural effusions or pericardial effusions were noted. No obvious changes were seen in the spleen, pancreas, adrenals, kidneys or lymph nodes.

The preoperative radiological FIGO staging was IIIC. The MDT recommendation was for primary debulking surgery (PDS).

Due to advanced FIGO stage disease at presentation with bilateral massive tumors involving both ovaries and the uterus, neither fertility‐sparing surgery nor cryopreservation of ovarian tissue could be considered. It was explained preoperatively that a fertility‐sparing approach would compromise survival and not be in her best interest. Fertility was not a priority for this patient at the time. She was consented for ultra‐radical ovarian debulking surgery to target R0 in a single attempt.

**FIGURE 1:**
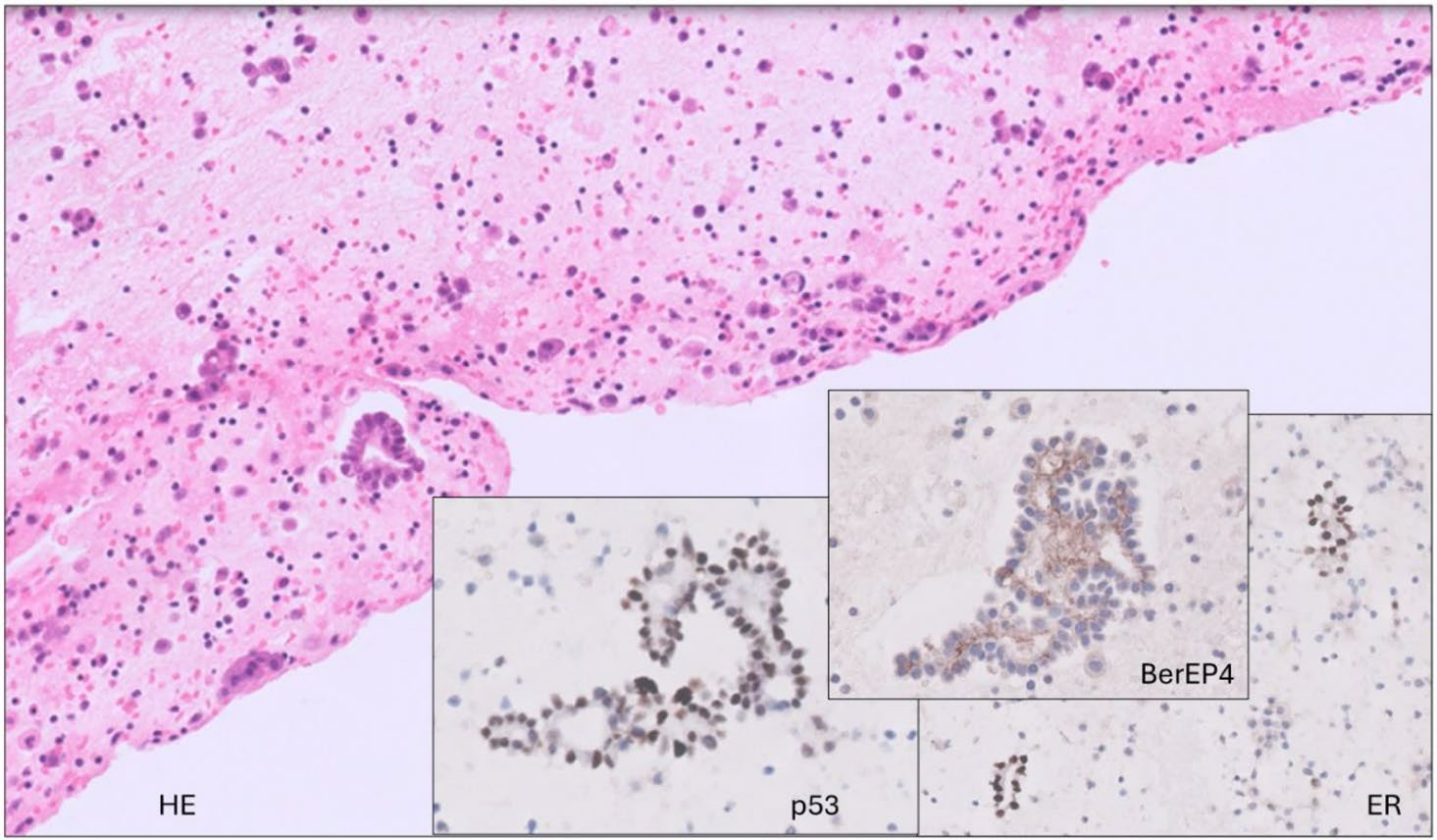
Cell block from ascitic fluid showing small papillaroid groups of epithelial cells with no overtly malignant features. These were expressing BerEP4, ER and showed wild pattern of staining for p53, HE (20×); BerEP4 (60×), p53 (60×) and ER (40×).

Prior to the planned surgical date, the patient was admitted for an ultrasound‐guided drainage of ascitic fluid as she was increasingly symptomatic and struggling to mobilize. Two liters were drained, with cytological examination showing neoplastic cells arranged in small papillaroid groups and no obvious high‐grade malignant features cytomorphologically or immunohistochemically. They showed diffuse strong staining for WT1, membranous staining for BerEP4, mostly diffuse strong staining for ER, a wild pattern of staining for p53 (Figure 1) and were negative for calretinin, consistent with a borderline or low‐grade ovarian serous tumor.

Pre‐operatively, a VTE risk assessment was carried out in line with OUH trust guidelines. Due to malignancy, major surgery and expected post‐operative immobility, our patient was planned to start prophylactic Dalteparin (5000 units) post‐operatively, with mechanical thromboprophylaxis used during (Flowtrons) and immediately after (TED stockings) the procedure.

## 4 Differential Diagnosis

The raised Ca‐125 as well as imaging were highly suggestive of ovarian cancer. Cytological analysis confirmed an ovarian neoplastic lesion in the peritoneal space with a differential diagnosis of SBT or LGSC. Both SBTs and LGSCs show a wild pattern of staining for p53 differentiating these tumors from much more aggressive and common HGSCs [10]. Cytological examination of ascitic fluid in the context of epithelial ovarian tumors is helpful in the differential diagnosis of low‐ and high‐grade tumors. However, definitive diagnosis of SBTs and LGSCs depends on identifying tumor invasion which is only possible on histological specimens with preserved tissue architecture. The final diagnosis of LGSC was possible only on histological examination of surgical specimens with demonstration of invasive tumor.

## 5 Treatment

Prior to the planned surgical date, the patient was encouraged to maintain a baseline fitness and improve nutrition as part of a prehabilitation programme. She received extensive counseling regarding the extent of surgery, length of hospital admission and long‐term implications of certain surgical steps, including fertility considerations, the probability of an irreversible stoma and lifelong antibiotics in the event of splenectomy. Cancer specialist nurses (CNSs) provided essential support for the patient throughout her clinical course. The stoma nurses were involved preoperatively.

The final surgical plan was agreed at a surgical MDT including the gynecology oncology and colorectal teams.

### 5.1 Surgical Procedure

**FIGURE 2:**
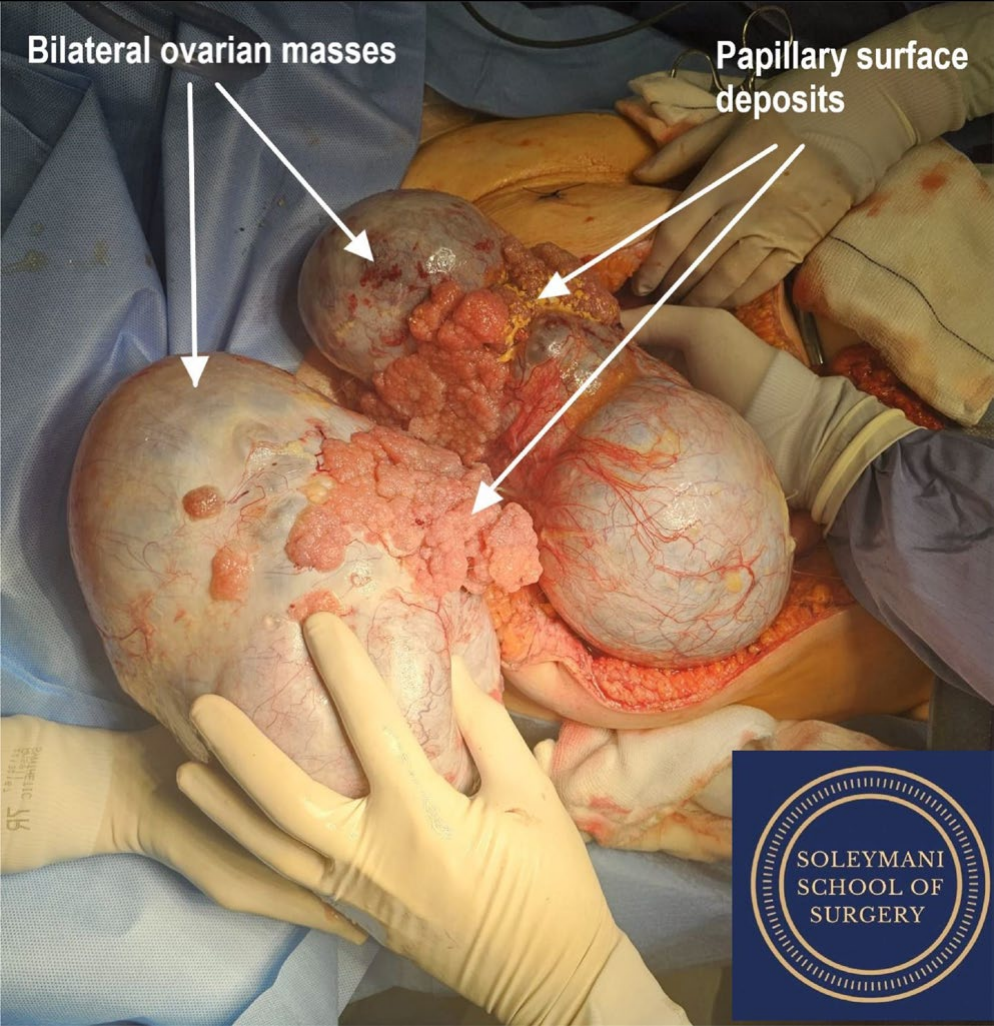
Massive bilateral ovarian tumor, with papillary surface deposits.

Prior to incision, the patient was cleaned, draped and catheterized. The legs were placed into a modified Lloyd‐Davies position to avoid well‐leg compartment syndrome and femoral nerve neuropraxia [11] with Flowtrons on which were monitored throughout the procedure. A midline xiphi‐pubic laparotomy was performed, with the abdomen opened in layers by handheld diathermy and Lahey forceps. Bilateral massive complex ovarian masses were identified with large exophytic lesions on the surface (Figure 2). Bilateral salpingo‐oophorectomy was performed to remove the enormous ovarian masses and facilitate access to abdominal and pelvic structures.

The large and small bowel were fully run to establish the extent of disease. Access to the side wall and bilateral mobilization of the descending and ascending colon was achieved via the Toldt line to the splenic and hepatic flexures respectively (Mattox and Cattell‐Braasch maneuvers). The small bowel and associated mesentery, ascending colon, transverse colon, descending colon, duodenum, stomach and omentum were all normal. The rectosigmoid colon was inseparable from the uterine body and likely invaded by tumor. The appendix appeared abnormal. Extensive peritoneal disease was found over the right diaphragm and the right abdominal and pelvic peritoneum.

Next, the right round ligament was opened, allowing development of the para‐rectal, para‐vesical and Latzko spaces. The right ureter was identified and a sling was applied. The right infundibulopelvic ligament was skeletonized and divided, whilst the right ureter was kept in view. The anterior division of the right internal iliac artery was tied 3 cm below the bifurcation of the right common iliac. The Okabayashi space was then developed with care to the ureters, which were kept lateral. Full ureterolysis was performed. The above steps were repeated on the left side.

Recto‐sigmoid resection was necessary as the recto‐sigmoid colon was inseparable from the uterine body. The sigmoid was divided using a GIA 80 mm stapler. The total mesorectal excision (TME) plane was developed, and the inferior mesenteric vessels were ligated. We proceeded to perform a Hudson retrograde hysterectomy with pelvic and bladder peritonectomies, following the 10‐step technique for en‐block resection of the pelvis [12].

**FIGURE 3:**
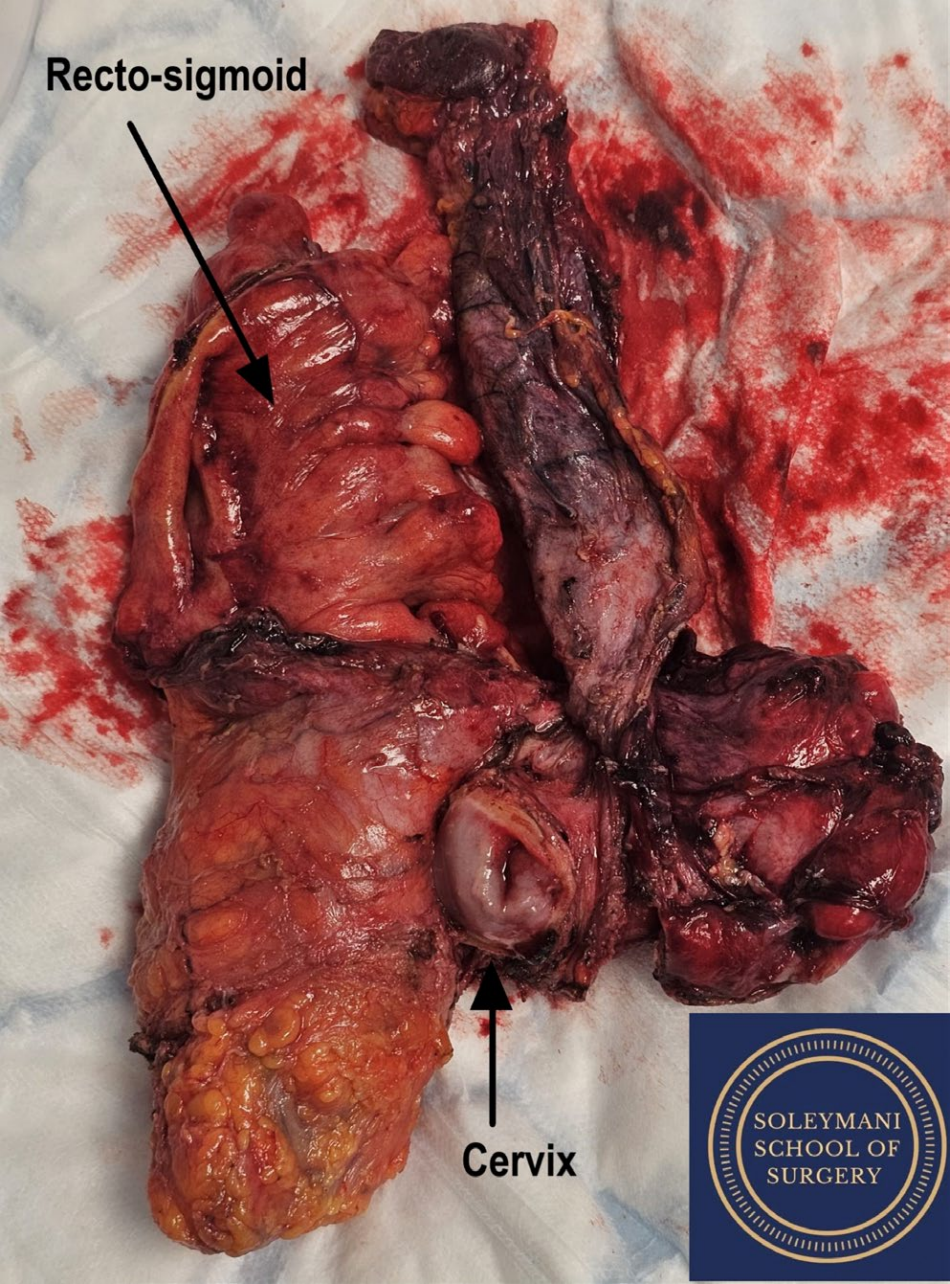
En‐block specimen including cervix, uterus, recto‐sigmoid colon, bladder and pelvic peritoneum.

Anterior colpotomy was performed and the rectovaginal septum was opened. The rectum was skeletonized and divided using a contour device. The en‐block specimen containing the cervix, uterus, bladder and pelvic peritoneum and the rectosigmoid was removed (Figure 3).

The para‐aortic and pelvic lymph nodes appeared normal.

A radical, supra‐colic omentectomy with preservation of the gastro‐epiploic arcade was performed.

While the tail of the pancreas was free of tumor, significant disease deposits were found over the spleen capsule and hilum, requiring splenectomy.

A right abdominal wall peritonectomy was performed to remove further tumor deposits.

Intra‐umbilical tumor deposits and a possible sister Marie Joseph nodule had also been noted, so the umbilicus was excised.

A type 3 liver mobilization was carried out, allowing full exposure of the right diaphragmatic surface. The triangular, right and left coronary ligaments were divided to expose the bare area. The hepatocaval ligament and Makuuchi ligaments were then divided to expose the retrohepatic inferior vena cava and adrenal gland, with the liver rotated in the left upper quadrant. The gallbladder, porta hepatis and liver were all normal.

Right diaphragmatic peritonectomy was performed to remove extensive diaphragmatic deposits, sparing the diaphragmatic muscle (type II diaphragmatic surgery) [13]. In a previous study, we have shown that in patients with FIGO stage IIC–IV ovarian cancer, diaphragmatic peritoneal disease extends to the muscle in 30% of cases, but this study did not include LGSC cases [14]. A 3 mm defect was identified on the right hemidiaphragm during this process and was repaired using 1/0 PDS. A suction Valsalva technique was applied to prevent pneumothorax, with post‐operative chest X‐rays and chest physiotherapy arranged to help monitor and mitigate any subsequent effects.

Finally, an appendicectomy was performed as the appendix appeared abnormal.

Robinson drains were inserted in both the right upper abdominal region, to drain the peripancreatic space, and in the right lower abdominal region, to drain the pelvis. A washout of the pelvis was subsequently performed. Ureters were observed to be vermiculating with no evidence of injury or devascularization. Urine remained clear throughout the procedure. The vaginal vault and pedicles were checked to confirm hemostasis.

Rectus sheath closure was achieved using 3× 1.0 PDS sutures tied together. Interrupted Vicryl was then used to close the adipose layer, and skin closure was completed using staples.

The end colostomy was matured to the left abdomen, and a colostomy bag was applied.

The estimated blood loss was 600 mL.

The postoperative plan included: use of TED stockings, prophylactic Dalteparin dose for 28 days (to start 6 h post procedure) and intravenous Co‐amoxiclav. Chest X‐rays (CXRs) were planned for days 1, 2 and 3 following surgery to check for pneumothorax, given the 3 mm diaphragmatic defect repaired following right diaphragmatic peritonectomy. The urinary catheter was to remain in situ for at least 5 days to rest the bladder following peritonectomy. Serum amylase and fluid amylase from the upper abdominal drain were to be analyzed on days 3 and 5 post‐operatively to monitor for pancreatic insult.

### 5.2 Histopathology Results

**FIGURE 4:**
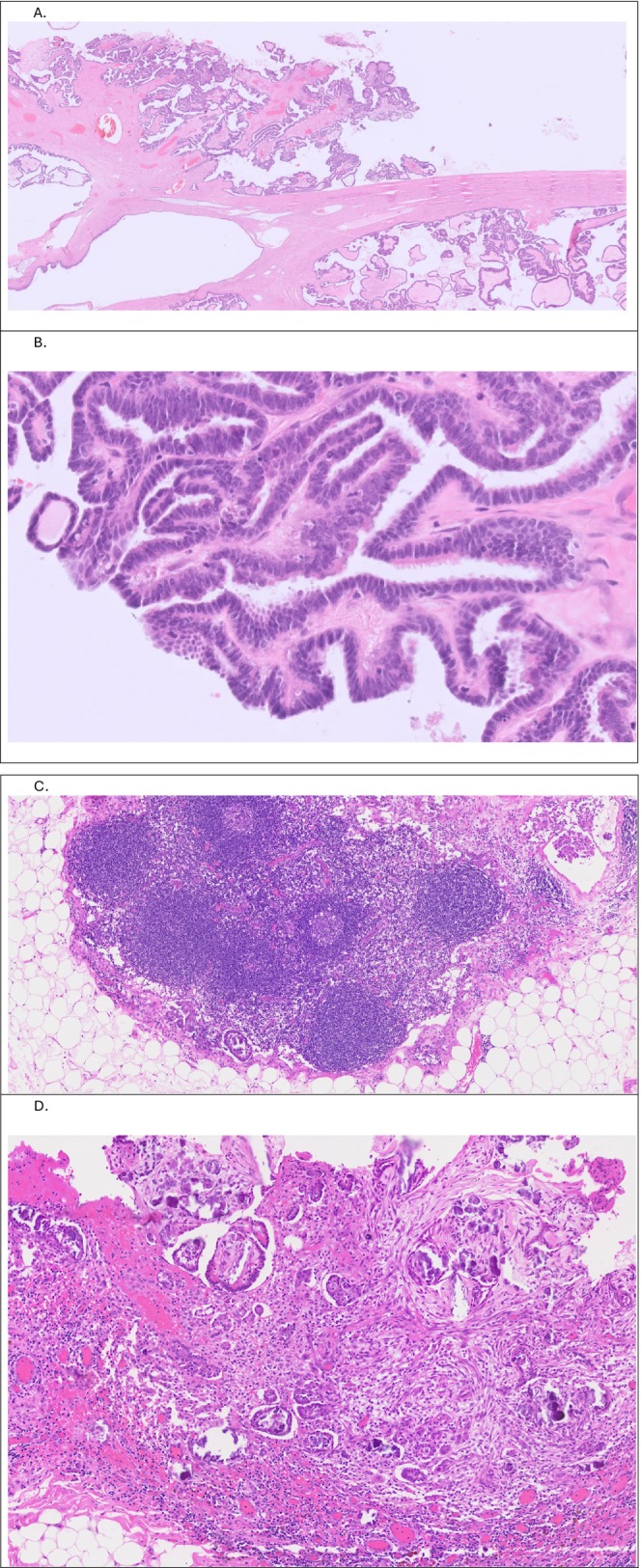
(A) Ovarian mass with tumor deposit on the surface, HE (2×); (B) Low grade nuclear atypia, occasional ciliated cells (arrow) and virtually no mitotic activity, HE (40×). (C) Lymph node metastases—papillary groups of tumor (arrows) in the subcapsular sinus in a lymph node from the paracolic fat, HE (10×). (D) Invasive deposits (arrows—small papillae in clear lacunar spaces) in the parietal peritoneum, HE (10×).

Surgical specimens submitted for histological examination included the uterus, cervix, bilateral ovarian masses and Fallopian tubes, rectosigmoid, spleen, omentum, appendix, umbilical lesion and pelvic and extrapelvic peritoneal tissue. The ovarian masses were intact but showed surface deposits. Microscopically, the ovarian tumors exhibited features of SBT with minor (~5 mm) micropapillary areas but no invasion. There were multiple peritoneal and omental deposits above and below the pelvic brim, a small number of which were invasive. Microscopic lymph node metastases were identified in 2 nodes—one in the mesocolon and one in the omentum. The tumor was classified as LGSC based on histological evidence of invasion (Figure 4A–D) and staged as FIGO IIIB based on macroscopic extrapelvic deposits with lymph node metastases. Intraoperative peritoneal washings contained neoplastic cells similar to those analyzed in ascitic fluid drained preceding the surgery (Figure 1).

## 6 Postoperative Recovery and Follow‐Up

During the ward round on day 1 post‐surgery, the patient was pale, dyspnoeic and tachypnoeic. Observations: HR 125 bpm, BP 80/69 mmHg, O_2_ sats 95% on 5 L, with desaturations to low 90s on room air. Her NEWS score was 10. Table 1 shows relevant blood results.

**TABLE 1:** Key blood results from the pre‐operative assessment, D1 post‐operatively during the diagnosis of the pulmonary embolism and D6 during the diagnosis of the retroperitoneal bleed.

Investigation	Pre‐operative assessment	D1 post‐operatively (PE)	D6 post‐operatively (retroperitoneal bleed)
Hemoglobin (g/L)	131	130	59
White blood cell count (×10^9^/L)	8.57	24.34	
Neutrophils (×10^9^/L)	4.98	19.90	17.91
Platelets (×10^9^/L)	515	303	489
Prothrombin time (s)	10.4	14.3	10.4
INR	1.0	1.4	1.0
Activated partial thromboplastin time (s)	28.2	34.4	34.2
Fibrinogen (g/L)	—	—	5.9
Albumin (g/L)	—	22	17
Urinary pregnancy test	Negative	—	—

**FIGURE 5:**
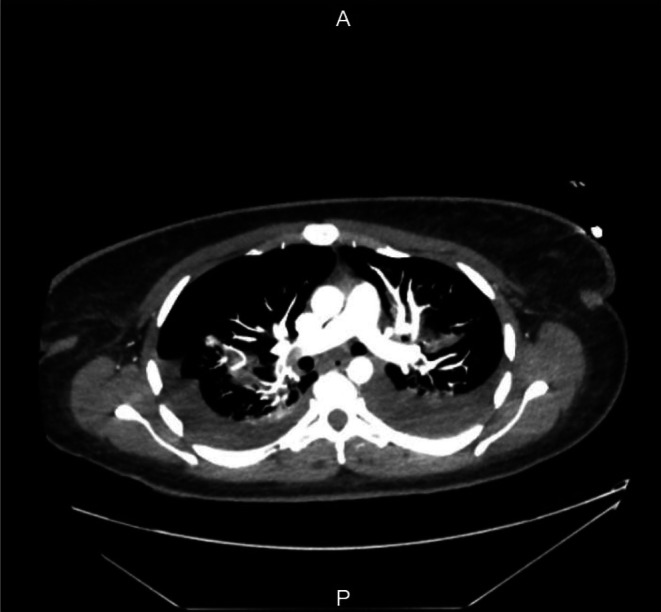
CTPA performed day 1 postoperatively, showing submassive bilateral pulmonary emboli, involving the right main pulmonary artery and extending into the right upper and lower lobar branches.

A CT pulmonary angiogram (CTPA) revealed bilateral submassive pulmonary emboli (PEs), involving the right main pulmonary artery and extending into upper and lower lobar branches (Figure 5). The left lower lobe segmental pulmonary arteries were also affected. A large right pneumothorax and bilateral moderate pleural effusions were present. Patchy airspace opacification was noted in the middle lobe and lingula, suggestive of pulmonary oedema or infection. Routine anticoagulation commenced 6 h post‐operatively (5000 units Dalteparin) was increased to therapeutic dosing (15,000 units). A bed‐side ECHO showed no right heart strain. The patient was transferred to Intensive Care (ICU). A multidisciplinary meeting with Radiology, Hematology and ICU colleagues agreed that drainage of the pneumothorax could follow therapeutic anticoagulation, so it was not a priority as it should resorb in the following days and did not cause the patient's respiratory failure. Therapeutic anticoagulation was administered in split doses for 24 h then as a single dose of 15,000 units. It was agreed that thrombectomy could be done should she fail to improve. This event was likely provoked by underlying malignancy and prolonged immobility associated with surgical intervention, with the only other risk factor being a raised pre‐op BMI (32). Her long‐haul travel from Australia had been over a month prior, and the patient had stopped smoking approximately 1.5 years before this event. A retrospectively calculated Khorana score was 2, for gynecological malignancy and raised white cell count (Table 1).

**FIGURE 6:**
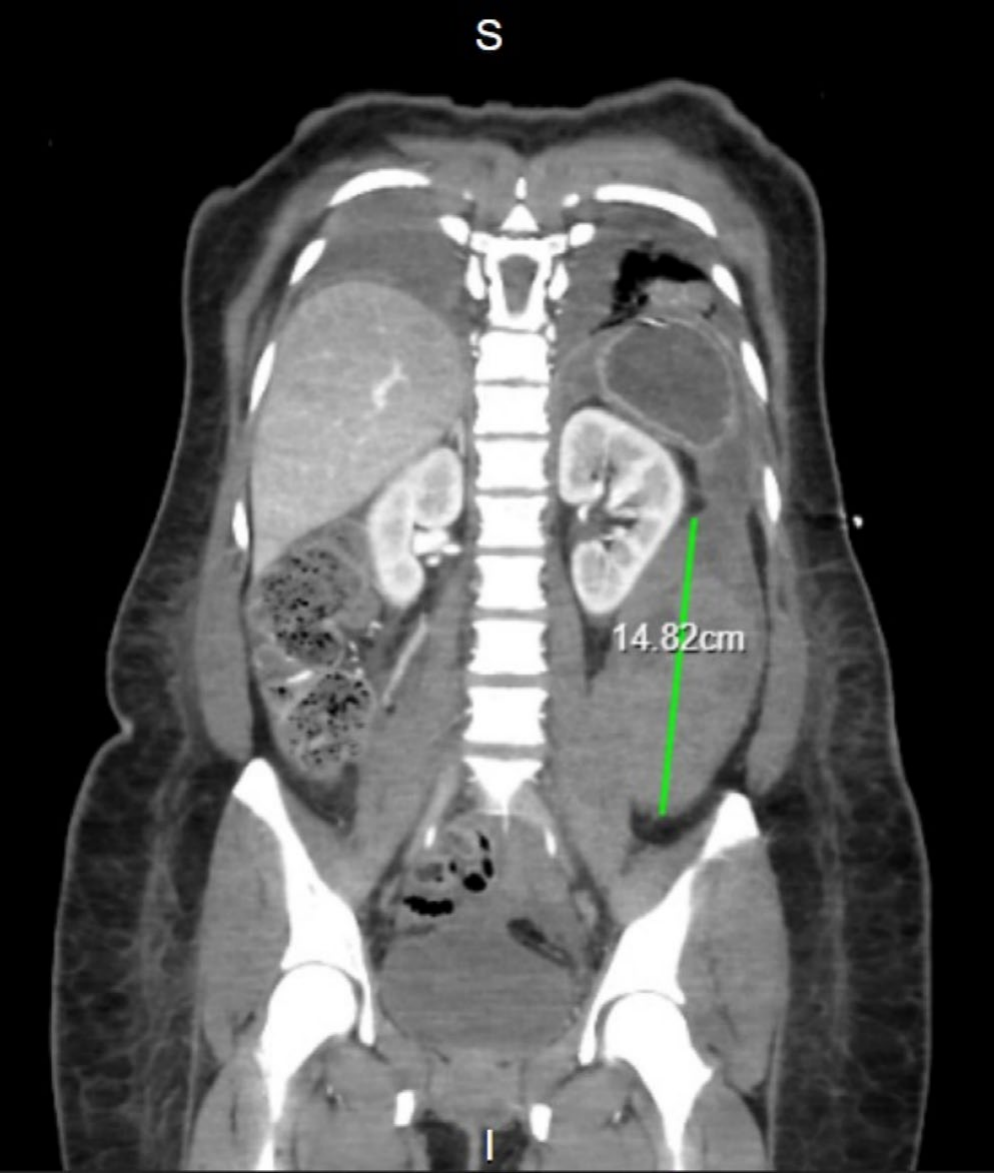
CT abdomen and pelvis performed on day 6 postoperatively, identifying a large left retroperitoneal haematoma, medial to the left iliopsoas muscle and inferior to the left kidney, in keeping with a haematoma, with no evidence of active bleeding.

Daily CXRs were ordered to monitor the pneumothorax and effusions, monitoring re‐expansion of the lung. On day 6 post‐op, the patient reported left flank pain, and an Hb drop (79–59 g/L) over 48 h was noted. A CT abdomen and pelvis and repeat CTPA were performed. The CTPA showed a significant reduction in the PE and right pneumothorax. However, a 9.2 × 6.5 × 14.8 cm left retroperitoneal collection was identified medial to the left iliopsoas muscle and inferior to the left kidney, suggestive of a haematoma without active bleeding (Figure 6). Clotting tests showed a mildly raised APTT (34.2 s) consistent with therapeutic Dalteparin. The patient was transfused 3 units of red blood cells, increasing Hb to 83 g/L the following day. Hematology input suggested reducing Dalteparin doses to 5000 units (prophylactic). A repeat CT after 48 h showed stabilization of the haematoma. A Doppler Ultrasound Scan of the lower limb veins showed no DVT. The Dalteparin dose was gradually increased in split doses (5000 units BD for 24 h, then 6000 units BD) to therapeutic. She started Apixaban 5 mg BD prior to discharge with planned hematology follow‐up.

Throughout the above, the patient was also experiencing a protracted ileus and delayed stoma function, which resumed on day 14 post‐surgery. She required TPN for 7 days. She experienced superficial wound dehiscence, which was managed conservatively. During her inpatient stay she received regular input from physiotherapy, dieticians, stoma nurses and the CNS team.

Following the splenectomy, the patient was started on prophylactic antibiotics (penicillin V), and was scheduled for routine vaccinations (pneumococcal, meningococcal ACWY conjugate, meningococcal B and influenza vaccines) via her GP.

Following discharge she was referred to medical oncology and has received 5 out of 6 cycles of Carboplatin, Paclitaxel and Bevacizumab, with maintenance Bevacizumab planned after her 6th cycle.

## 7 Discussion

Pulmonary embolism is the second leading cause of death in patients with cancer [15]. The association between malignancy and thrombosis is well known and was first described by Trousseau in 1896 [16]. Patients with ovarian cancer are at particularly high risk, with one study reporting a pre‐treatment incidence of up to 27% [17]. This is due to the Virchow triad of stasis: mechanical obstruction, hypercoagulability from tumor‐derived procoagulants and dehydration related to third spacing, and endothelial injury [18]. Intra‐ and post‐operative factors such as surgical duration and immobility further increase risk. The mortality of patients who develop PE is ~15% [19] with 75% of deaths occurring within 1 h and the remainder within 48 h [20].

Our patient had massive bilateral ovarian masses, causing long‐term compression of the iliac vessels and likely development of thrombi. As our patient was diagnosed with submassive PE within 12 h post‐procedure, this may indicate that thrombi were already present and dislodged during surgery. Prompt diagnosis and treatment resulted in full recovery from a life‐threatening complication. Management of PE following extensive ovarian cytoreductive surgery is a challenge due to the concurrent risk of bleeding. Multiple treatment dilemmas were discussed by an MDT including Gynecology Oncology surgical, ICU, Hematology and Radiology teams. Firstly, it was decided that the treatment of PE took priority over the management of pneumothorax, which was likely to resolve spontaneously. A chest drain insertion would have delayed the administration of the treatment dose of Dalteparin. Secondly, it was deemed safe to administer therapeutic Dalteparin, albeit in split doses in the first 24 h. Thrombectomy was planned in the event of further deterioration despite therapeutic anticoagulation. Thrombolysis is not advised immediately post‐operatively due to the bleeding risk. The patient developed a retroperitoneal haematoma on day 6 post‐surgery, and was managed conservatively with extensive multidisciplinary input and careful Dalteparin dose adjustment.

The routine postoperative prophylactic Dalteparin regimen did not prevent thrombosis in this high‐risk patient. Some studies suggest pre‐operative screening with D‐dimer level [17, 21, 22] and Doppler ultrasound [17] prior to surgery to detect pre‐existing thromboses, although this is not included in international ovarian cancer guidelines. Another consideration could be pre‐operative administration of Low Molecular Weight Heparins (LMWH e.g., Dalteparin) in high‐risk patients [23].

Limitations of our case report detailing the unusual presentation of a rare tumor are the inability to generalize our findings and establish any cause–effect relationship. Data collection to national or international registries may help establish the best course of management for patients with rare tumors, in which randomized controlled trials are not feasible [24].

To conclude, we have described a case of advanced stage LGSC affecting a very young patient. Ultraradical cytoreductive surgery was performed in conjunction with the colorectal team and complete resection of all visible disease (R0) was successfully achieved—the only prognostic factor for survival in a relatively chemo‐resistant tumor. The patient had an intricate post‐operative course characterized by both submassive PE and retroperitoneal bleeding, managed through regular multidisciplinary input from ICU, Hematology, Radiology and Gynecology Oncology. We are delighted to report that our patient made a full recovery from surgery and has now completed cycle 5 out of 6 of adjuvant chemotherapy.

## Author Contributions

**Hannah Williams:** writing – original draft, writing – review and editing. **Sabina Ioana Nistor:** conceptualization, investigation, supervision, writing – review and editing. **Slaveya G. Yancheva:** investigation, writing – review and editing. **Nicholas Symons:** investigation, writing – review and editing. **Hooman Soleymani majd:** performing the surgery, conceptualization, investigation, supervision, writing – review and editing.

## Consent

The patient has consented to have her case written and published. This was done with knowledge of the process and possible outcomes.

## Conflicts of Interest

The authors declare no conflicts of interest.

## Data Availability

All data associated with the report is available as part of the article and no additional source data are required.
